# Compression-Assisted Adaptive ECC and RAID Scattering for NAND Flash Storage Devices

**DOI:** 10.3390/s20102952

**Published:** 2020-05-22

**Authors:** Seung-Ho Lim, Ki-Woong Park

**Affiliations:** 1 Division of Computer and Electronic Systems Engineering, Hankuk University of Foreign Studies, Yongin 17035, Korea; slim@hufs.ac.kr; 2 Department of Computer and Information Security, Sejong University, Seoul 05006, Korea

**Keywords:** NAND flash memory, P/E cycle, compression, adaptive ECC, RAID scattering, stripe log, parity

## Abstract

NAND flash memory-based storage devices are vulnerable to errors induced by NAND flash memory cells. Error-correction codes (ECCs) are integrated into the flash memory controller to correct errors in flash memory. However, since ECCs show inherent limits in checking the excessive increase in errors, a complementary method should be considered for the reliability of flash storage devices. In this paper, we propose a scheme based on lossless data compression that enhances the error recovery ability of flash storage devices, which applies to improve recovery capability both of inside and outside the page. Within a page, ECC encoding is realized on compressed data by the adaptive ECC module, which results in a reduced code rate. From the perspective of outside the page, the compressed data are not placed at the beginning of the page, but rather is placed at a specific location within the page, which makes it possible to skip certain pages during the recovery phase. As a result, the proposed scheme improves the uncorrectable bit error rate (UBER) of the legacy system.

## 1. Introduction

With advances in process geometry scale-down of flash memory, a rapid increase in the capacity of NAND flash memory chips has been achieved. The main reason for this rapid increase in capacity is the increase in the number of bits stored per cell. However, as this number increases, the inter-electron interference degree increases, resulting in an increase in the error rate of the flash memory [1,2]. In addition, as the program/erase cycle (P/E cycle) increases; that is, as the usage time increases, the physical characteristics of the cell deteriorate, and the error rate increases rapidly. This is a serious drawback of computer systems based on NAND flash memory storage devices.

The typical approach to rectify this short endurance and increase in errors is using error-correction codes (ECCs) [3,4]. NAND flash memory devices use the ECC module to create additional parity for data-error correction. The parity generated by the ECC module is stored in the out-of-bound (OOB) region within a page; a page is the unit of read and write operations. Therefore, ECC is used for error correction in page units. Although ECC parities can achieve error-correction gain, if there are more errors than an ECC can correct, they may have no choice but to return decoding failures, if used without any additional error-recovery scheme for the failed data. However, creating more ECC parity is a complex and difficult process because the ECC module is a type of hardware block within the flash memory controller. One useful approach for creating more ECC parities for specific data is to use lossless compression [5,6], where additional ECC parity is created and stored in the free area after compression of the data.

In contrast, as a complement to in-page ECC decoding failures, an error management method using another parity technique between outer-pages, such as RAID5, has often been applied. Many previous works show that parity with the RAID5 technique can reduce the page error rate [7,8,9,10]; however, it has also been shown to result in increased additional write operations and low space utilization owing to RAID parity management. That is, the RAID parity operation creates an additional write operation in the parity update process. From a space management point of view, the smaller the size of a cluster that has RAID parity, the larger the number of parities, resulting in a space overhead.

In this paper, we propose an enhancement scheme for an in-page ECC parity ability and outer-page RAID parity management with the help of lossless data compression. In the proposed system, incoming data are compressed through a compression module, and then, so-called adaptive ECC and RAID-scattering schemes are applied to this compressed data step by step. For in-page ECC improvement, ECC parity is created by applying ECC encoding only to the compressed data. Compression reduces the source length applied to ECC encoding, which results in lowering of the code rate.

The code rate is defined as a rate occupied by data in a codeword, so the lower the code rate, the higher the parity rate; thus, the error correction capability is improved by compressing data and applying ECC only to these compressed data.

After compression, data are then placed in each offset position within the page, in which the placement position is determined by its position in the RAID stripe.

We refer this placement scheme as RAID-scattering scheme. Since the data are compressed and occupy a smaller area than the page, each datum occupies only a specific part of the page, and the rest becomes a non-use area, and the value of the non-use can be set a known value. As a result, the specific positioning of each compressed data creates a region where non-use regions overlap each other in a stripe. Owing to the overlapping of the non-use area, the overhead of restoring data in RAID is reduced, because when data are restored with RAID parity algorithm for the error-occurred page in the RAID stripe, the non-use area of the error page does not need to be restored.

As a result, some pages can be skipped during the restoration process.

In other words, it results in more pages being recovered by each RAID parity, and parity overhead is reduced at the same level as the RAID reliability. In addition, this paper describes a FTL architecture, a FTL management scheme and the associated metadata for parity logging that can efficiently update and manage RAID parity on NAND flash devices. The scheme proposed in this paper is an extension of our previous work [11] that only considered in-page ECC management. This paper extends and highlights a combination of in-page ECC management and outer-page parity management, as well as detailed metadata structure of RAID parity management.

The organization of this paper is as follows. In Section 2, background and related work are presented. The proposed adaptive ECC and RAID-scattering scheme is described in Section 3, and experimental results are shown in Section 4. Finally, Section 5 concludes this paper.

## 2. Background and Related Work

In this section, the background of this research area is first described, which includes basics of flash memory and flash memory-based systems. Then, the related work is described.

### 2.1. NAND Flash Memory-Based Storage Devices

**Figure 1:**
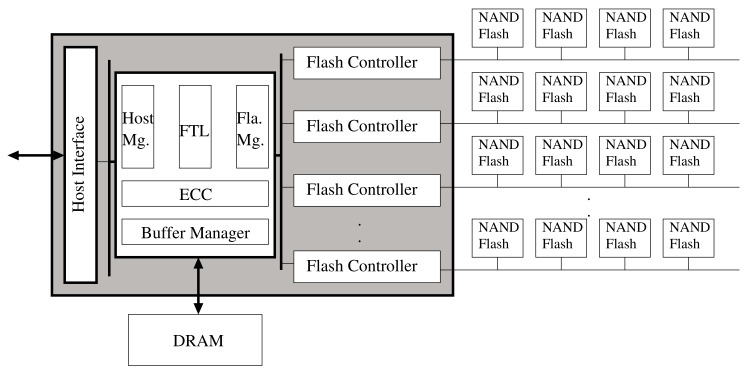
Basic architecture for NAND flash-based storage devices.

Figure 1 describes typical NAND flash-based storage devices. The basic hardware architecture of NAND flash memory-based storage devices consists of a high-performance controller, DRAM main memory, and an array of NAND flash memory chips. NAND flash memory is a nonvolatile semiconductor storage device that is formed by integrating a floating gate-based semiconductor device. The main components include a page, which is a read and write unit, and a block, which is an erase unit. The page sizes are mainly about 4 KB, 8 KB, and 16 KB, and dozens of pages are collected to form a block. There are three internal commands used for NAND flash memory: read, program, and erase. The read and program commands transfer data to and from the flash chip, and a data transfer unit is a page. Actually, the erase command does not transfer data and works in block units. A client device can make only two types of requests, namely, a read and a write request. The read request is related to the read command and the write request is related to the program command.

Currently, triple-level cells (TLC) are the most commonly used NAND flash memory. It stores three bits per cell, where each of the three bits of data in a TLC flash cell are either programmed or erased, which means the cell has a total of eight different states from 000 to 111. In contrast, a single-level cell (SLC) and a multi-level cell (MLC) store one bit and two bits per cell, respectively. With several states, the quantized voltage level of memory cells decides the value of the cell. The growth in the number of states per cell raises interference between states as the quantized decision levels of the cell start moving close between adjacent states, which results in detection errors. Recent advances in 3D lamination technology make it easier to secure cell-to-cell spacing compared to conventional 2D methods, resulting in a slight increase in P/E cycles compared with those of conventional 2D.

There are two crucial points regarding NAND flash memory. One, if the data are written to a flash memory, write operations should be preceded by erase operations internally. Second, the basic units of erase and write operations are different from each other. To overcome the unit mismatch between write and erase operations and to efficiently use flash memory, special software layers called flash translation layers (FTLs) have been developed [12,13,14,15,16,17]. Beyond single internal operational issues, other works have focused on the parallelism issue within embedded storage devices to speed them up [18,19,20,21,22,23].

### 2.2. Error Correction Codes for Flash Memory

**Figure 2:**
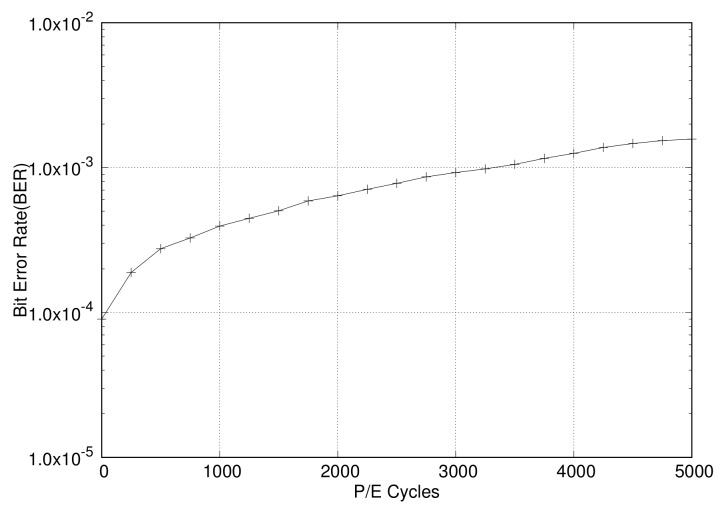
Evaluated bit error rates according to P/E cycles for TLC NAND flash memories [24].

The first-generation NAND flash memory was much simpler than the current generation. However, over time, the storage capacity of NAND flash memory has increased by moving to smaller geometries, and also storing more bits per cell. This required a much better error-correction algorithm to ensure data integrity for NAND flash devices. The P/E cycle value is about 100,000 for SLCs, 10,000 or less for MLCs, and 1000 or less for TLCs. As error rates increase rapidly, the usage of flash devices is emerging as a major issue. The advent of TLC flash and even smaller geometries will further increase the error-correction requirements in NAND flash. Figure 2 shows the raw bit error rate (RBER) for the P/E cycle of the TLC NAND flash memory [24]. As shown in the figure, when there are thousands of P/E cycles, the RBER is so high that it is known to reach the lifecycle limit.

As a method for recovering such errors, ECC methods [3,4,25,26,27,28], such as BCH and LDPC, are embedded in the NAND flash memory controller, which can cover page-level errors for programming and retrieving data. The BCH codes form a class of cyclic error-correcting codes that are constructed using polynomials over a finite field. An arbitrary level of error correction is possible, and it includes efficient code for un-correlated error patterns. Low-density parity check (LDPC) codes are considered one of the best choices for current flash memory controllers owing to their excellent error-correction capabilities. LDPC code decoding is an iterative process with the decoding input as certain probability information, which leads to a dependency of LDPC error correction on the accuracy of the input information [4]. ECCs for flash memory storage systems have been widely studied in both BCH and LDPC. Ref. [3] addresses applications using BCH ECC coding driver on a Linux platform, and many other chip designers have presented BCH bit error coding implementations. In contrast, LDPC receives attention from the industry, as there have been many presentations on LDPC in flash summits in the recent past [25,26]. In [27], a LDPC decoding strategy optimized for flash memory was proposed. Tanakamaru et al., have shown that LDPC codes can improve SSD lifetimes by 10 [28]. K. Zhao presents three techniques for mitigating an LDPC-induced response time delay [4].

### 2.3. Parity with RAID

While ECC can prevent data errors inside a page, many other approaches for preventing data errors outside a page exist. RAID [29] enhances the reliability by using redundant data. RAID creates an additional parity for clustered data to ensure error recovery. Cluster means a group of units associated with parity data. In a general RAID system, this unit is a disk; however, inside an SSD, it can be a block or a chip. The parity is generated by using bitwise exclusive-or operations between blocks that belong to the same cluster, and thus, parity updates usually require two block reads and two block writes in traditional disk-based storage systems. However, in flash-based storage devices, owing to the out-of-place characteristic of flash memory, the approach of RAID techniques is different; they mainly focus on reducing write requests for designing flash-based RAID schemes.

Previous works show that parity achieved by using the RAID5 technique can reduce the page error rate compared with ECC. However, the parity overhead of the RAID architecture has its own limitations, as a lot of reads for existing data must precede new parity calculations. There are several previous works that have discussed ways to reduce the parity overhead. Lee [8] retains the parity blocks in the buffer memory, postponing parity writes until the idle time, which can reduce read operations and write response time; however, system crash at partial stripe write points cannot be recovered as parity updates are postponed until a full stripe is written. Im [30] generates partial parity for partial stripes so that partial write is possible; however, additional non-volatile RAM (NVRAM) hardware is required as partial parity is maintained in NVRAM. Kim [10] employs variable size striping, which constructs a new stripe with data written to portions of a full stripe and writes parity for that partial stripe without any additional hardware support. However, this approach generates too much partial parity, and therefore, degrades write performance and space utilization.

## 3. Compression-Assisted Adaptive ECC and RAID Scattering

This Section describes, first, the adaptive ECC and RAID-scattering scheme, followed by the metadata structure and operation of FTL for RAID parity management as an implementation issue.

### 3.1. Compression-Assisted Adaptive ECC

**Figure 3:**
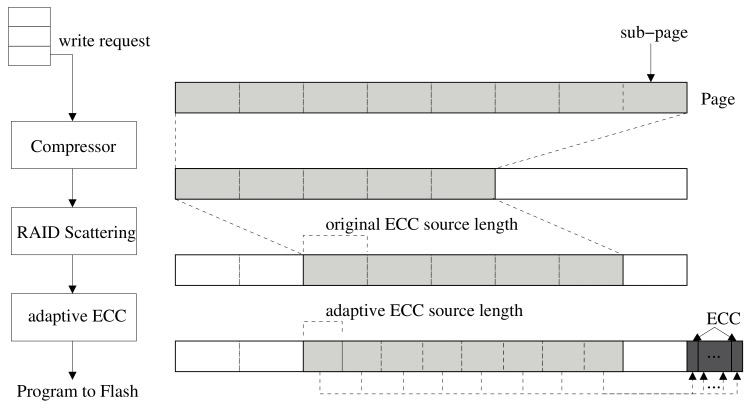
Data write procedure with compression-assisted adaptive ECC and RAID scattering.

From the perspective of storage efficiency, data compression is an effective way of enabling a high-density storage system, and a significant amount of user’s data in storage media could be compressed according to [31,32]. We incorporated this compression mechanism in an attempt to provide the enhancement of error recoverability. In this system, the incoming write request goes through two steps before being programmed into flash cells, that is, RAID scattering and adaptive ECC with compression, as shown in Figure 3. At first, the incoming data are compressed using the compressor module. Then, the compressed data are placed at some position within the page buffer, and the unused region caused by compression is filled with 1 s because the value of 1 s is electrically stable for flash memory.

In general, the read and write units of NAND flash memory are page units; however, a page is divided into sub-pages for ECC encoding/decoding. The size of a page is usually 4 KB, 8 KB, or 16 KB; each page can be composed of four to eight sub-pages. For example, if a page has an 8 KB data area and a spare area for 1 KB of ECC, it consists of eight 1 KB sub-pages and a space area for 128 bytes of ECC. The write operation method of this system is as follows. When write data are sent from the host, the data are compressed by the compression module. If the compressed and reduced size is not larger than the sub-page size, it is not necessary to store data in more than one sub-page. The compressed data are moved to a specific position within the page by the RAID-scattering module. Thereafter, the ECC is generated by the adaptive ECC module.

Compression modules have already been applied to improve the ECC function [5,6]. However, in existing works, unused areas due to data compression are used to store additional redundancy. In this system, in contrast, we use ECC encoding and decoding only for the compressed region, leaving the unused region intact. This method can be applied with little modification to existing ECC engine modules and encoding schemes. The procedure is as follows. After compression, the size of the compressed data is divided by the number of sub-pages, i.e., *N*, to obtain the source size of ECC encoding. *N* is denoted as the number of ECC operations for each page, which is fixed for the implementation of legacy flash controller. This ensures that the source length of the ECC encoding is smaller than the existing sub-page size. With the reduced source by compression, the variable-sized ECC [33] is applied to generate ECC parity, and the generated ECC parity size does not change. The ECC parity size remains the same, and the code rate indicating the ECC parity relative to the source length is lowered, indicating a higher error correction rate.

The instance of the data-writing process and ECC-generation process is described in Figure 3. As shown in the figure, it is assumed that one page, with size 8 KB, is composed of eight sub-pages. The 8 KB of data transmitted from the host were compressed using the compression module, resulting in 5 KB of compressed data. It means that three sub-pages do not need to store data. These data are moved to the third sub-page position by the RAID-scattering scheme. The RAID-scattering scheme is discussed in the next subsection. As the data are reduced from 8 KB to 5 KB by compression, one ECC encoding size of 5 KB data is 640 bytes, which is 5 KB/8. This represents a reduction in code rate, which means that the ECC-correction capability is improved. Because ECC encoding is an error-correction function for data stored in a page, the adaptive ECC scheme improves the in-page error-correction function.

### 3.2. RAID Scattering

If data are compressed by the compression module, the size of the compressed data would be smaller than the original size, so it occupies only a specific part of the page. In our system, when the compressed data are stored in the flash memory, it is not stored from the beginning of the page but in a specific position in the page. This position is determined according to the stripe number where the page is located in the stripe. Therefore, RAID scattering is defined as positioning compressed data in a specific location on the page according to the position in the corresponding stripe.

**Figure 4:**
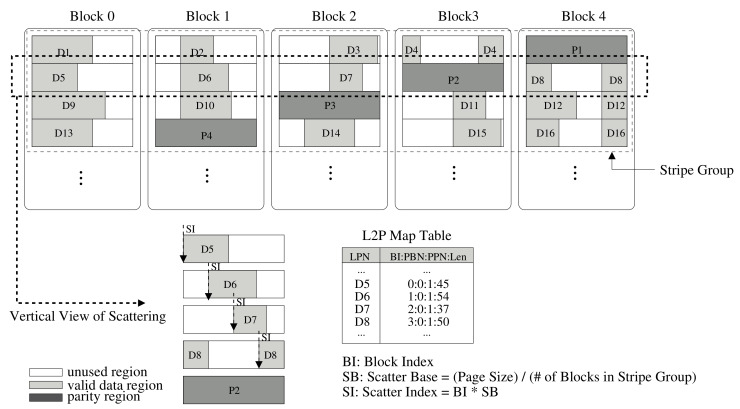
Compression-assisted RAID scattering architecture.

For instance, the architecture of RAID scattering with a stripe size four times larger than the page size is described in Figure 4. In the RAID configuration, SG (Stripe Group) is a group of blocks belonging to a stripe, and the notation BI (block index) is a block number in the stripe group; SB (Scatter Base) is a page size divided by the number of blocks in a stripe, and SI (Scatter Index) is the offset for the placement of each compressed data unit in a stripe group, calculated as SB multiplied by BI. For each write request, the compressed data are placed at the offset value SI calculated by SB and BI. If the data do not exceed the page size starting from a specific position, i.e., SI, all of the compressed data can be stored on the page, as appropriate. If the amount of data exceeds the limit of the page, the remaining data are placed from zero offset at the page, in circular manner. For example, as shown in Figure 4, if the number of blocks in a stripe group is four and the page size is 8 KB, data D5 are placed at offset zero in the page, data D6 are placed at offset 2 KB, data D7 is placed from offset 4 KB, and data D8 is placed offset by 6 KB in the page, respectively. As a result, all compressed data can be placed at different positions within their stripes by the RAID scattering scheme. Then, RAID parity is created by xor operation of all data in the stripe. In Figure 4, P2 is the RAID parity created through the xor operation of D5, D6, D7, and D8.

In our system, if data cannot be recovered by in-page ECC, it will be recovered via the outer-page error recovery, i.e., RAID parity. In an existing RAID system, when attempting to recover an error page by using the RAID parity of the stripe group containing the page where the error occurred, all pages in the stripe group must be read to perform the xor operation. However, in the RAID scattering system, it is not necessary to read all pages of the stripe group. Instead of reading all the pages in the stripe group, it is only necessary to read the pages that overlap the actual valid area of the compressed data of the page where the error occurred. For instance, in Figure 4, let us assume errors occur during the read of data D5, which means error is not recovered by in-page ECC. In this case, to recover data with RAID parity, the RAID scattering system reads only D6 and D8 and performs the xor operation to recover D5. D7 does not overlap with D5, and hence reading for the recovery operation is unnecessary.

To identify overlap information of pages in a stripe group, the length of the compressed data is added in a mapping-table in the FTL metadata. The compressed length is an important parameter to determine which pages are overlapped for recovery, as well as to decompress data for the read operation. The added length information is depicted in Figure 4. The length of valid data is represented by the actual length of the compressed data. In our implementation, the length field has a compact size for reducing metadata space overhead. For instance, a 12-bit field is required to represent a maximum length of 4 KB page, and a 13-bit field is required to represent a maximum length of 8 KB page. So maximum 3 bytes per entry is required for that.

Using the method of scattering within a stripe group has an effect of reducing the association of pages with each other within a stripe group. In other words, it is possible to increase the size of the stripe group for the same level of association. That is, if a RAID parity in the existing RAID system covered a stripe group with stripe size four, then another RAID parity in the RAID scattering system can cover a stripe group with stripe size eight. This results in reducing the overhead of RAID parity.

### 3.3. Metadata Structure and Parity Management

**Figure 5:**
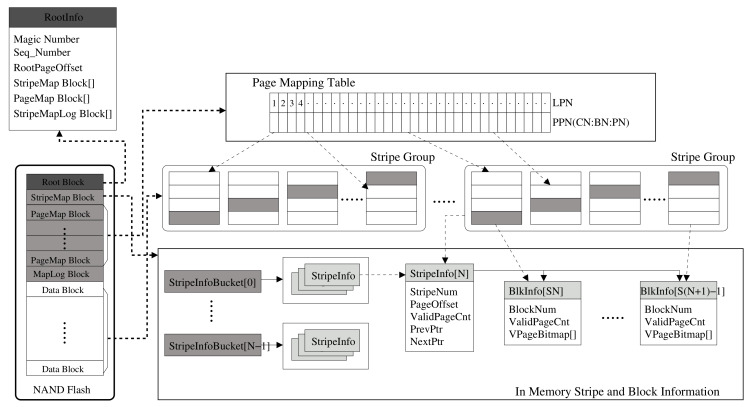
Software metadata structure for RAID and parity management.

The design objective of metadata structure is to reduce flash write operations for metadata and parity data of RAID5 architecture. For this, the metadata changes are updated as a logged fashion with metadata log structure, and parity is always flushed once at all writes of the corresponding stripe is completed. The designed metadata structure for a RAID stripe and parity management is shown in Figure 5, where this metadata are present in the FTL layer. The left part of the figure shows the in-NAND metadata snapshot structure, which is composed of the root block, StripeMap block, PageMap block, and MapLog block. For the root block, either flash memory block 0 or 1 is used as the pivot root block. At first, block 0 is assigned to the root block. The root block contains information of metadata blocks, and it is mainly used to find the metadata blocks at system boot. The last updated page of the root block contains information called RootInfo that contains the location of the blocks where the metadata are stored in Flash memory. Each time the metadata block changes, RootInfo is updated on the one page of the root block as in a log. When the update reaches the last page of the current root block (e.g., block 0), the root block changes to block 1 and the update continues.

The StripeMap block contains the stripe information for each stripe. Those stripe information is managed by three types of data structures: BlkInfo, StripeInfo, and StripeInfoList. The BlkInfo contains the status information for each data block, and StripeInfo has the status information for each stripe group. The size of the stripe group is the number of data blocks included in the stripe. For example, if the stripe size is *S*, one stripe group consists of *S* blocks. StripeInfoList is a hash data structure with a bucket from 0 to (S×N), where *N* is the number of valid pages in the stripe group.The stripe information includes stripe number, valid page count for the stripe, page offset, and so on. Each bucket has a list of dual-connected stripe groups with the same valid number of pages. It is used to select the free stripe group for the next write and the victim stripe group to perform when garbage collection (GC) is needed. The PageMap block contains page-mapping table information. Each entry in the page-mapping table is an array of physical page numbers (PPNs). The last part of the metadata is the StripeMapLog block, which is used to log the metadata changes information for data writes. The StripeMapLog block is important for our metadata log architecture. The in-NAND flash metadata blocks represent the latest version of the snapshot, while the main memory contains the latest metadata that has changed since the last snapshot. The metadata changes are written as a logged manner so that the latest metadata can be maintained.

**Figure 6:**
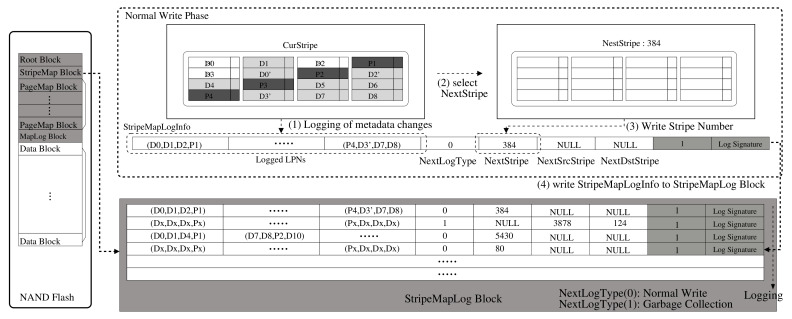
Stripe map log operations for RAID and parity management.

A metadata log operation is illustrated in Figure 6. Each metadata log is composed of one StripeMapLogInfo structure, and the StripeMapLogInfo consists of an array of logical addresses of the corresponding stripe group, which represent logical-physical mapping information. A stripe group is a group of blocks grouped together to form a RAID stripe. The stripe group is at the block level; however, the parity for the stripe is at the page level within the group. For example, if a stripe is composed of four blocks, parity is generated based on three pages with the same page offset in the group. The logging strategy is for metadata logging not for data logging. So, whenever data write occurs, the data are just written to flash memory, while its metadata change is not flushed immediately. Each time a new write occurs, new data are allocated to a new page within the stripe group, and the LPN of the corresponding PPN is logged to StripeMapLogInfo in memory. The updated metadata information, that is, the logical addresses for the written data in this stripe group, is just maintained as the StripeMapLogInfo structure information in memory until all pages of the stripe group are used for writes. Once it is filled with a certain amount of metadata updates, the stripeMapLogInfo is flushed to the last available page in the StripeMapLog block. When the StripeMapLog block is full of loaded pages, thus, no longer available for logging, the next available block area is needed. Before allocating a new StripeMapLog block, metadata information in the main memory is synchronized to the flash to be the latest snapshot, and then, a new StripeMapLog block is allocated. After this process, the logging mechanism described above is repeated.

In addition to the metadata logging, full stripe parity update operation is designed and implemented. In our system, parity for the stripe is retained in a buffer memory until full stripe write is done. The full stripe write means that all of the data for the corresponding stripe is written. Since parity is updated in memory buffer with the incoming data of the stripe, it does not need to read old parity and old data to update parity. The parity is just written when a full stripe write is completed. Since partial parity is maintained in memory, it can be lost with power failure. The metadata log information is used during the recovery process of the partial parity from power failure. When power loss occurs during the parity is in partial state retained in buffer, that partial parity can be recovered by scanning the logged information.

### 3.4. Power Loss Recovery Step of Metadata Log and Partial Parity

The metadata log and partial parity retained in memory can be lost from power failure; however, it can be recovered by recovery process. Since the metadata changes after the snapshot are logged to the StripeMapLog block, we can recover metadata changes by scanning the MapLog blocks. For the paritis, almost all parity data are stored in flash memory as a status of parity for full stripe write; the actual portion to be recovered is just the partial parity of write data for the corresponding stripe before power loss.

The metadata log and parity recovery steps are as follows. This step is power loss recovery step, not error recovery step. At the first step, flash storage tries to find the latest RootInfo by scanning Root block. In the second step, it recovers the metadata snapshot that consists of StripeInfoList, StripeBlkInfo, BlkInfo, and page mapping table by retrieving StripeMap and PageMap blocks. In the third step, the metadata changes, that occur after the snapshot, were stored in the StripeMapLog blocks, so they are recovered by reading page by page and updating each information. The last step to be recovered is the metadata changes that were losted due to the power loss are recovered. Those were just maintained in-memory StripeMapLogInfo so were not flushed to StripeMapLog block. Metadata changes of the StripeMapLogInfo can be recovered by scanning the corresponding stripe group itself, and we can identify the stripe group number from last valid page in the StripeMapLog Block since we record the number of NextStripe group at last valid StripeMapLog. So, the mapping changes of the NextStripe group can be recovered by scanning the spare region of each page. If the page has valid logical address in the spare region, which means the data are stored safely so that its metadata should be updated. If not, the data are not stored safely and the pages are abandoned. For the scanning of each stripe page, if all the pages of the stripe have valid logical address, which means all the data for the stripe are stored safely, the corresponding parity is also safe. If some pages do not have valid logical addresses, which means not all the data in this stripe are valid, then there is no valid parity for this stripe. For this stripe, each valid page is retrieved and parity is calculated with those. It is the partial parity that is lost by power failure. Therefore, we recovered partial parity for this stripe.

## 4. Evaluation

In order to prove the validity of the proposed RAID scattering and adaptive ECC, we implemented the proposed schemes inside FlashSim [34,35] that is widely used in the storage system evaluation. This open source simulator includes the simulated NAND flash controller and NAND flash memories that can be set for page size, block size, etc. The simulator also implements well-known FTLs and NAND flash operations, including read, write, and erase, as well as garbage collection. We have added the RAID parity management, compression-based adaptive ECC, and RAID-scattering schemes in this simulator. The simulation configuration set for performance evaluation is as follows. The page-level FTL was used for an FTL mapping management scheme, in which 8 KB was used for page size, and a 1 KB sub-page was used. The compression module uses the zlib [36] compression module. The BCH ECC model is used with 1024BCH16, which is an ECC module that can correct 16 bits per 1024 bytes. That is, 1024BCH16 is applied for the 1 KB sub-page. For the bit error generation and simulation, the BER is sampled and an error is injected according to its P/E cycles. The samples from BER versus P/E cycle data are taken from a previous study on NAND flash devices [24]. The simulated BER sample is depicted in Figure 2.

### 4.1. Analysis of Adaptive ECC and RAID Scattering

To evaluate the performance of the proposed system in different user environments, various IO traces, such as filesystem workloads, database workloads, web server access data, and media streaming IO, are collected by developers reflecting the real human behavior. The IO traces are collected and categorized as F-1, F-2, F-3, F-4, and F-5 according to the application’s characteristics. Among those IO traces, F-1 and F-2 are text-dominant file system and database traces, F-3 and F-4 are media-dominant streaming application traces, and F-5 is a mix of IO traces.

**Figure 7:**
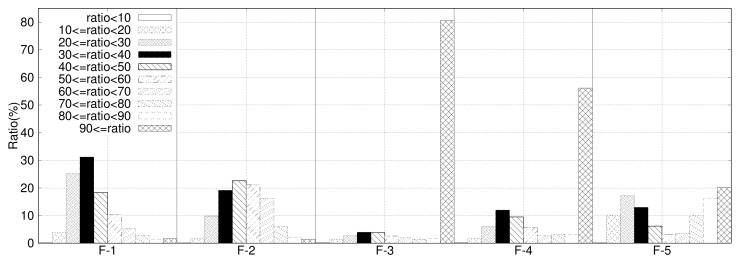
Distribution for compressions for each dataset.

We have measured the impact of compression for various IO traces. We applied page-based data compression using the zlib [36] compression module and measured the compression ratio for each page. The compression ratio distribution for IO traces from F-1 to F-5 is shown in Figure 7. As shown in the figure, compression ratios are distributed differently among all IO traces. For general filesystems and databases, large chunks of data are compressed to less than 50% of the original data, which means much of data for general filesystems and databases are highly compressed. The portion of text-like data in filesystems and databases is large, and these types of data are generally compressed at a high compression rate. On the contrary, for web servers or media streaming applications, not much data are compressed, as depicted in F-3 and F-4. Media-like data, such as videos, images, and audio, are dominant on web servers, which are inherently compressed data, and hence, the compression rate is low, which results in low compression rate.

Based on the distribution for the data-compression rate of IO traces, we have evaluated the adaptive ECC scheme and the RAID-scattering scheme. For a fair evaluation, we have simulated IO operations over 10 million times and checked errors for each trace, as bit errors occur rarely. We measured the uncorrectable bit error rate (UBER) [1] for RBER as a performance metric by increasing the P/E cycle from 0 to 5000 for each experiment of each configuration. For UBER a page cannot be recovered by using the error-recovery scheme.

**Figure 8:**
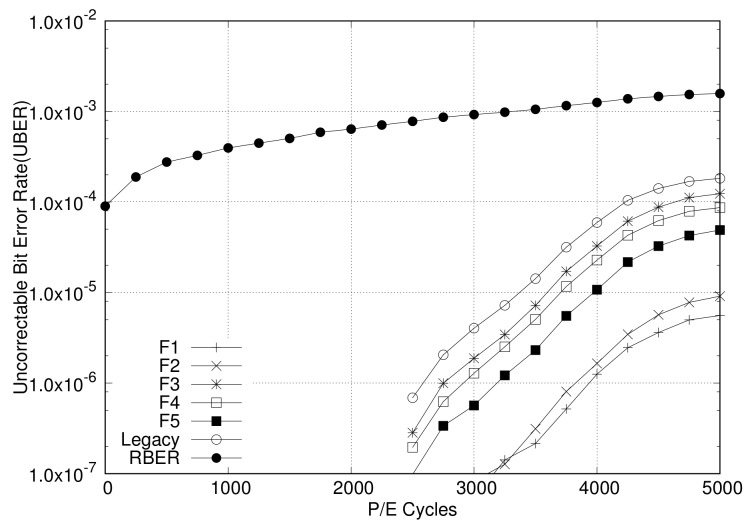
Adaptive ECC scheme for IO traces from F-1 to F-5 in comparison with legacy.

At first, the adaptive ECC scheme is evaluated to show its own performance contribution to error recovery. This is achieved by comparing with the original ECC scheme, that is, fixed-sized ECC encoding. The results of the error-recovery ability for adaptive ECC are plotted in Figure 8. The *x*-axis represents P/E cycles, and the *y*-axis represents UBER on a logarithmic scale. Thus, the result error rate after ECC decoding is presented as UBER. As per the results, the lower the UBER, the better the error-correction capability. As shown in Figure 8, the adaptive ECC scheme results in better UBER over legacy ECC for all IO traces, which means that the adaptive ECC scheme shows a better performance than the fixed ECC scheme in the correction of bit errors. The reason of this increase is the reduction in valid data from compression. From the figure, we can also identify that the performance gap increases for increasing P/E cycles. It means that more errors can be covered by adaptive ECC as more bit errors occur. Among IO traces, F-1 and F-2 result much better UBER than other traces. Because F-1 and F-2 achieve higher compression rates than others, these give smaller source sizes for ECC decoding. On the contrary, F-3 and F-4 show little performance improvement in comparison with that of the original ECC owing to inefficient compression.

**Figure 9:**
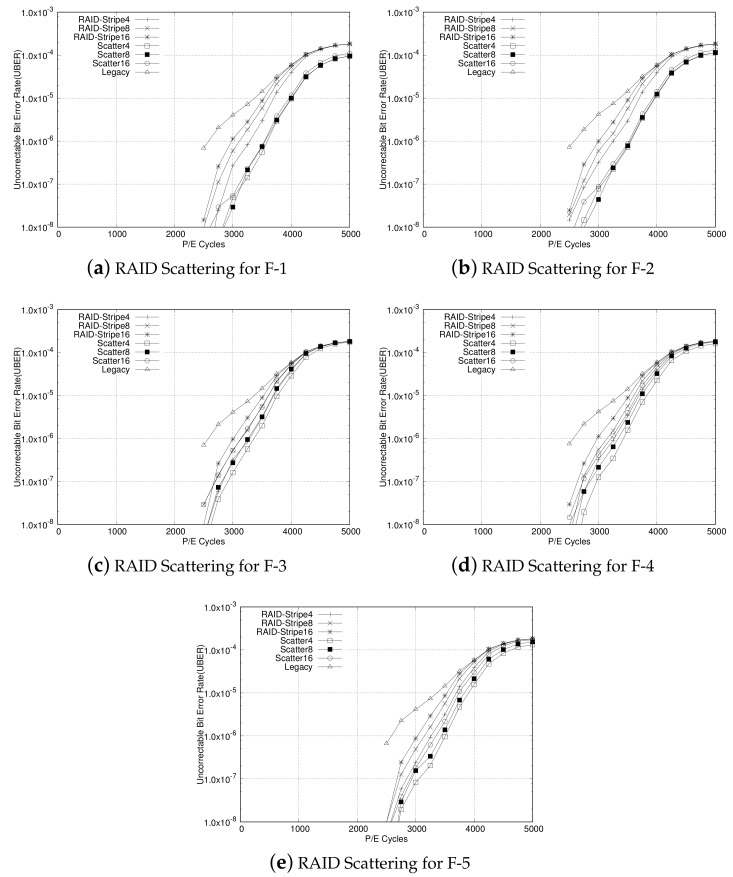
Experimental results for RAID scattering in comparison with original RAID with various stripe sizes. In accordance with P/E cycle, UBERs of RAID Scattering and original RAID are estimated along with stripe sizes four, eight, and 16.(**a**) RAID Scattering for F-1, (**b**) RAID Scattering for F-2, (**c**) RAID Scattering for F-3, (**d**) RAID Scattering for F-4, (**e**) RAID Scattering for F-5.

Next, the RAID-scattering scheme is compared with the original RAID scheme having the RAID5 parity model. For this evaluation we have simulated the original RAID and RAID scattering with varying stripe sizes of four, eight, and 16. For all RAID and RAID-scattering configurations, UBER is evaluated as the P/E cycle increases from 0 to 5000. The experimental results are plotted in Figure 9. In all the figures, in accordance with the P/E cycle, RAID-Stripe4, RAID-Stripe8, and RAID-Stripe16 represent UBER for the original RAID configuration with stripe sizes four, eight, and 16, respectively, while Scatter4, Scatter8, and Scatter16 represent UBER for the RAID-scattering scheme with stripe sizes four, eight, and 16, respectively. We have also plotted Legacy UBER to compare results with the RAID-scattering scheme, as well as the RAID system. The Legacy shows the original uncorrected page error rate of the original fixed ECC scheme.

From Figure 9a,b,e, we identify that RAID scattering gives UBER enhancement over the legacy RAID system for F-1, F-2, and F-5. This is due to the high compression ratio of these traces. In the RAID system, the error-containing page can be recovered using the parity page and other data pages within the corresponding stripe. If more than one of those pages also has an uncorrected page error, the error-containing page fails to be recovered. As data are compressed and scattered within a stripe in the RAID-scattering system, there is less probability for overlap between error-containing pages than in the original RAID system. This results in less UBER for RAID-scattering systems for traces F-1, F-2, and F-5. For F-1 and F-2, we identify that RAID scattering with all stripe sizes give less UBER than legacy RAIDs with even smallest stripe size, that is, four. This means that even if the stripe size is large and the parity covers a wider range, the error-correction capability is better than of the original RAID with a smaller stripe size. However, for IO traces with lower compression rates such as F-3 and F-4, as shown in Figure 9c,d, there is no big difference between UBER of the RAID-scattering scheme and UBER of the existing RAID scheme. As compression is less effective for F-3 and F-4, the number of pages to be recovered in the corresponding stripe has an error rate that does not differ much from that of the original RAID scheme. Even so, the figure shows that there is some performance improvement for each P/E cycle.

**Figure 10:**
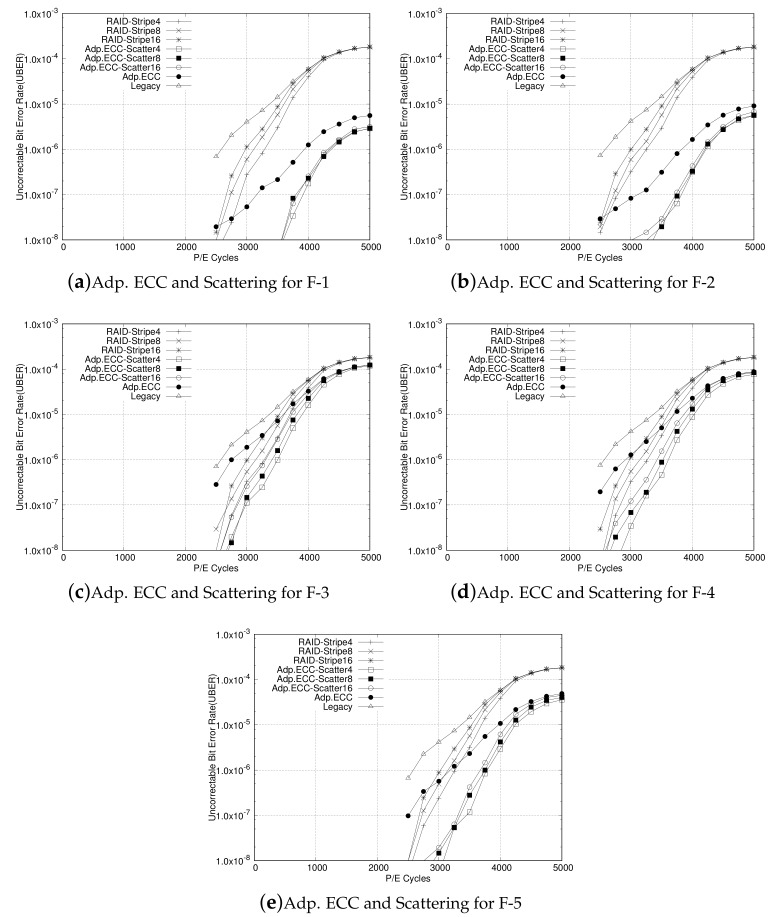
Experimental results for RAID scattering added by adaptive ECC in comparison with the original RAID with various stripe sizes four, eight, and 16. In accordance with P/E cycle, RAID-Stripe4, RAID-Stripe8, RAID-Stripe16, Adp. ECC-Scatter4, Adp. ECC-Scatter8, Ada. ECC-Scatter16 show UBER with stripe sizes four, eight, and 16, respectively. (**a**) Adp. ECC and Scattering for F-1, (**b**) Adp. ECC and Scattering for F-2, (**c**) Adp. ECC and Scattering for F-3, (**d**) Adp. ECC and Scattering for F-4, (**e**) Adp. ECC and Scattering for F-5.

Finally, the RAID-scattering scheme with the adaptive ECC is compared with the legacy RAID system. In this experiment, each IO trace is modeled as compressed, encoded with adaptive ECC, and stored as scattering within the stripe in the RAID system. Like for the previous evaluation, we have simulated those by varying stripe sizes with four, eight, and 16. For all RAID and RAID scattering with adaptive ECC configurations, UBER is evaluated as the P/E cycle increases from 0 to 5000. The results for traces from F-1 to F-5 are plotted from Figure 10a–e. Similar to the results of the RAID scattering only scheme, all figures show plots for UBER for RAID-Stripe4, RAID-Stripe8, RAID-Stripe16, Adp.ECC-Scatter4, Adp.ECC-Scatter8, and Adp.ECC-Scatter16 configurations in accordance with the P/E cycle. From the figures, we identify that RAID scattering with adaptive ECC shows a lower UBER than that of legacy RAID, as well as RAID-scattering scheme. For all cases, it definitely results in less UBER than legacy RAID systems, regardless of the compression rate of the IO data. Another noticeable point is that the UBER for RAID scattering with adaptive ECC is less than that of the legacy RAID even though the stripe size is larger, which implies that the RAID-scattering scheme can enlarge the stripe size for the RAID system configuration with a higher reliability. In other words, by reducing parity overhead, RAID scattering can reduce write overhead at the same reliability level as it has less parity portion.

**Figure 11:**
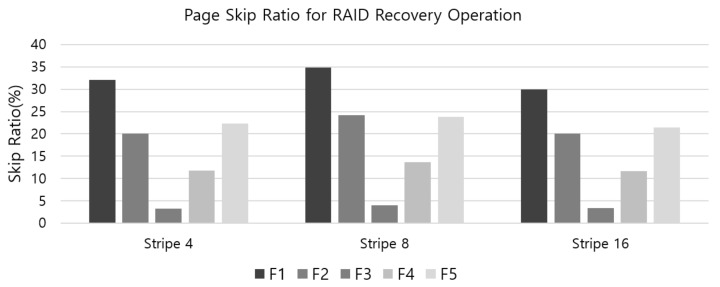
The skip ratio of pages when restoring error-occurred page in the stripe in the RAID scattering scheme.

To see how much pages can be skipped during the recovery stage in RAID scattering scheme, we have measured the number of pages to be skipped from the recovery operations for the error-occurred page. It was also done with the RAID configuration as stripe size four, eight and 16, respectively, and for each configuration, the five IO traces were performed. Figure 11 shows the ratio of pages skipped during each restoring error-occurred page in the RAID scattering scheme. In the figure, five IO traces are depicted as a group to the same stripe size level, and thus the *x*-axis represents groups of five IO traces according to stripe size level, and the *y*-axis represents the skip ratio. Since the skip ratio represents the ratio of pages that need not be read during recovery; the higher the skip ratio is, the better the recovery rate is as well as the shorter the recovery time is. From the results, it can be inferred that since the compression ratio is high as in the traces F-1 and F-2, the skip ratio is high, which results in reduced UBER. This is an effect of the RAID scattering scheme. On the other hand, the skip ratio of F-3 is low; there is little effect on RAID scattering, which results in little enhancement for UBER.

### 4.2. Analysis of Metadata and Parity Overhead

The adaptive ECC and RAID-scattering schemes provide an effective error management method that can be achieved by reducing the size of the valid data regions on a page. However, additional overhead is required to support this operation. There is an overhead in storing and managing the size of the compressed data stored in each page. Specifically, a space for recording the compressed data size of the corresponding page is required for each page entry of the FTL mapping table. It takes about 14 bits or 3 bytes per entry. The necessary metadata size can estimated according to page size, block size, and capacity of flash device. For 128 GB flash device, whose page size and block size is 8 KB and 1 MB, respectively, the amount of estimated metadata blocks is about 119–122 blocks which includes two root blocks, four StripeMap blocks, 112 PageMap blocks and 1–4 MapLog blocks. The size is about 120 MB. If the capacity of flash device is double, the amount of estimated metadata blocks is almost double of previous one. For 512 GB flash device, whose page size and block size 8 KB and 3 MB, respectively, the amount of metadata blocks is about 155–158 blocks including two root, two StripeMap, 150 PageMap, and 1–4 MapLog blocks, and whose size is around 237 MB. From the estimation we identify that it is the PageMap block that occupies the largest weight in metadata, and it increases as the number of pages increases. Since we use about 7 bytes per entry of the pagemap to manage the physical address of compressed data, it is considerably larger than the PageMap which does not manage data compression. On the other hand, the additional metadata blocks for stripe management consumes less than 10 blocks for hundreds of GB of flash devices, which is not much overhead.

To see the metadata overhead in the aspect of NAND flash IO, we analyzed the overhead of the metadata logging IO operation. The overhead of the metadata log management is mainly composed of two parts. The first is page write operations that write StripeMapLogInfo, which is a log of metadata changes for the corresponding stripe group. The StripeMaplogInfo is written to a page in the StripeMapLog block whenever the page’s current stripe group is used for data write. The other part involves the flushing of whole metadata snapshot, which occurs when all the pages of StripeMapLog block are exhaused. The whole metadata snapshot includes PageMap, StripeMap, and RootInfo. To evaluate the metadata log overhead, we have generated random write operations to the simulator and measured the number of written pages for metadta logs, which include logging of StripeMapLogInfo and the flusing of the entire metadata. Among the metadata log overhead, the duration of writing metadata snapshot is dependent of the number of StripeMapLog blocks. If we have several StripeMapLog blocks, the duration of flushing metadata snapshot is longer since more StripeMapLogInfo can be written to the StripeMapLob blocks. Therefore, the experiment was conducted by changing the number of StripeMapLog blocks from one to four.

**Figure 12:**
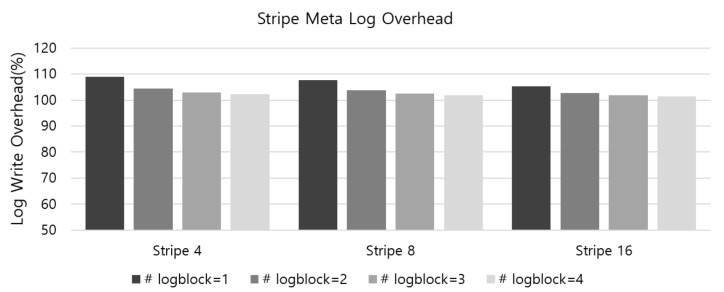
The results of metadata log overhead for the metadata log system for RAID5 architecture with stripe size four, eight, and 16, respectively.

Figure 12 shows the results of metadata log overhead for the implemented metadata log system for RAID5 architecture with stripe size four, eight, and 16, respectively. As shown in the figure, metadata log overhead is almost 10 percent if the RAID5 configuration is set with four stripe size and 1 StripeMapLog block. However, the overhead decreases as the stripe size increases and the number of StripeMapLog block increases. As the stripe size increases, the number of pages included in the one stripe group increases, and thus the frequency of flushing the StripeMapLogInfo log increases. In addition, if the number of StripeMapLog blocks is increased, the number of flushing of whole metadata snapshots is reduced. From the results, it can be seen that when the number of StripeMapLog blocks is increased by four or more, the overall metadata log overhead can be reduced to 5% even if the capacity of flash device is hundreds of GB.

Next, to analyze the parity overhead, we have measured the number of parity writes for random write requests while varying average request size from 4 KB to 128 KB. The experiments are done with two RAID5 configurations, that is, full stripe parity scheme and conventional RAID5 scheme. The full stripe parity scheme implemented in this paper was compared with the conventional RAID5 scheme, in which parities of RAID5 are updated whenever a write request occurs for the corresponding stripe. Those configurations are also set to four, eight, 16 stripe size, respectively.

**Figure 13:**
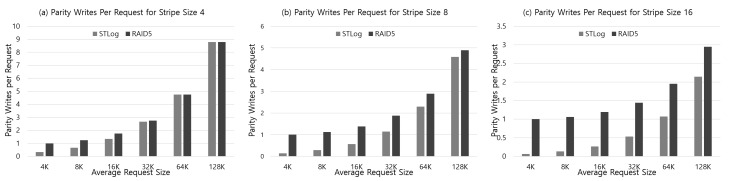
Result of the number of parity writes per request according to the change in the average request size, for each stripe size four, eight, 16, respectively.

The result of the parity overhead according to the average random request size is depicted in Figure 13, for each stripe size four, eight, 16, respectively. In the figure, the *x*-axis represents the average request size and the *y*-axis represents the number of parity writes per request. The number of parity writes per requests means the number of parities written for each request, and therefore, the lower the value, the smaller the parity overhead. Two RAID configurations, STLog and RAID5, are depicted, where STLog stands for the full stripe parity scheme and RAID5 for the conventional RAID5 scheme. As shown in the Figure 13a, if the stripe size is four, there is little difference between STLog and RAID5. There is lower parity overhead for small requests, and parity overhead becomes similar as the request size increases. However, if the stripe size is bigger, i.e., eight or 16, the parity overhead is largely reduced with the STLog parity management scheme as shown in Figure 13b,c. It is due to the partial parity buffering of the full stripe parity scheme. Particularly, when the request size is smaller than the stripe size, the partial parity buffering applied in STLog is more effective since parity updates are frequently performed in the conventional RAID5 scheme. If the request size exceeds the stripe size, parity for full stripe write is created as it is, so STLog is no different from conventional RAID5.

### 4.3. Discussion about Compression Module

The adaptive ECC and RAID scattering is applied based on lossless data compression, and thus the overhead of the data compression module is a performance-deteriorating factor for NAND flash memory devices. Although the software zlib module is used in our system for data compression, the compression module requires a lot of CPU computation, and hence it is not suitable for practical use in software implementations. However, recent compression hardware [37] or accelerator [38] further increases the practical possibilities of lossless data compression. In particular, [37] describes that it supports gzip/zlib/deflate data compression with approximately 100 Gbps bandwidth. If such a compression module is included in the NAND flash controller, it is expected to be able to perform data compression with little effect to the performance degradation of other modules, although it will increase the cost. In this paper, we could not analyze the performance degradation effect by the compression module, but in the future, there is a need to analyze the performance of the overall system including the hardware compression module. This is our further work.

## 5. Conclusions

The density of flash memory devices has increased by moving to smaller geometries and storing more bits per cell; however, this generates a lot of data errors. The typical approach to check these increasing errors is the use of well-known ECCs; however, the current state of the ECC scheme it not on the same level with the evolution of errors of the current state of flash memory. The RAID technology can be employed for flash storage devices to enhance their reliability. However, RAID has inherent parity update overhead.

In this paper, we propose the enhancement for ECC ability for inside the page and RAID parity management for outside the page, with the help of lossless data compression. The compressed data are encoded with adaptively reduced source length, which increases the recovery ability of the ECC module. The adaptive ECC method can reduce the size of a source length in proportion to the compression ratio. As this reduces the source length to be encoded, the effect of lowering the code rate increases the error-correction rate. For the perspective of outside the page, the compressed data are placed at a specific position in the page. As the unused area in the page generated by compression can be used as a non-overlapping area in RAID scattering, it is effective during the RAID recovery stage. The unused area is spread out within the parity cluster, which reduces the error coverage range due to the possible skipping pages that are not overlapping with the error-occurred page. The experimental results show that adaptive ECC can enhance the ability of error recovery. Furthermore, the RAID-scattering scheme achieves a high reliability at the same RAID stripe level. As the next step of this study, we will apply compression, ECC, and RAID scattering modules as hardware modules to evaluate the actual overhead and performance impact. Therefore, a more rigorous comparison of the performance of the propose scheme versus others could be an important task to improve the completeness of the proposed scheme. Consequently, we set the more rigorous performance evaluations as our further work.

## Abbreviations

The following abbreviations are used in this manuscript:

ECC	Error Correction Code
RAID	Redundant Array of Independent Disks
SLC	Single-level cell
MLC	Multi-level cell
TLC	Triple-level cell
OOB	Out-of-Band
BCH	Block Hamming Code
LDPC	Low-Density Parity Check
SSD	Solid-State Disks
GC	Garbage Collection
FTL	Flash Translation Layer
LPN	Logical Page Number
PPN	Physical Page Number
SG	Stripe Group
BER	Bit Error Rate
RBER	Raw Bit Error Rate
UBER	Uncorrectable Bit Error Rate
NVRAM	Non-Volatile Random Access Memory
GC	Garbase Collection
